# Optimal dose of normal saline for confirming correct peripheral venous access with precordial Doppler ultrasonography in children

**DOI:** 10.1038/s41598-023-32578-5

**Published:** 2023-04-12

**Authors:** Ayaka Omori, Yuji Otaki, Motoi Tanaka, Mitsunori Miyazu, Sachiko Ohde, Taiki Kojima

**Affiliations:** 1 Department of Anesthesiology, Aichi Children’s Health and Medical Center, 426 Nana-Chome, Morioka-Cho, Obu, Aichi 478-8710 Japan; 2 Department of General Pediatrics, Aichi Children’s Health and Medical Center, Obu, Aichi Japan; 3 Graduate School of Public Health, St. Luke’s International University, Tokyo, Japan; 4 Division of Comprehensive Pediatric Medicine, Nagoya University Graduate School of Medicine, Nagoya, Japan

**Keywords:** Health care, Medical research

## Abstract

Precordial Doppler ultrasound technology can be utilized to confirm correct peripheral intravenous vascular (PIV) access in children during surgery. This study aimed to determine the minimally required dose of normal saline (NS) for confirming correct PIV access. Healthy children were randomly allocated to receive a 0.1 mL/kg, 0.3 mL/kg, or 0.5 mL/kg dose of NS injected via PIV access. Two independent raters judged the change in the recorded precordial Doppler sound test (S-test) before and after NS injection. Typically, rapid injection of NS increased the pitch of the heartbeat as the injection volume increased. Changes in blood flow velocity test (V-test) results were evaluated using a cut-off value of 1 cm/s. Both in the S- and V-tests, the detection rate of correct PIV access was lower with 0.1 mL/kg NS than with 0.3 mL/kg or 0.5 mL/kg. Logistic regression analysis showed that the positive results in both the S- and V-tests were significantly decreased with a 0.1 mL/kg NS; no significant difference was observed with a 0.3 mL/kg NS (reference dose: 0.5 mL/kg). These results suggest 0.3 mL/kg is the minimally required dose of NS for confirming correct PIV access. This study is registered with the University Hospital Medical Information Network (UMIN000041330).

## Introduction

Infiltration of a peripheral intravenous vascular (PIV) access can cause various skin tissue injuries in children. These injuries include severe consequences such as skin necrosis and compartment syndrome, which may require surgical intervention^1^. In addition, PIV access infiltration during general anesthesia results in inadequate delivery of anesthetic medications; this may lead to unexpected patient movement during surgery. Previous studies have reported an infiltration rate of 2–3% in children outside operating suites^2–4^. Generally, correct PIV placement can be identified based on clinical judgment (e.g., blood aspiration by a syringe, swollen appearance of skin by direct observation of the PIV insertion site, absence of free drop of infusion by gravity). However, these clinical judgments are often inconclusive and misleading. Surgical drapes covering the whole body of the children usually inhibit direct observation of the PIV insertion site. The blood cannot be aspirated even through correct PIV access because the small veins of children easily collapse under negative pressure. Therefore, pediatric anesthesiologists still desire an alternative method for confirming correct PIV access during surgery.

Our previous preliminary studies have shown that precordial Doppler ultrasound can confirm correct PIV placement by detecting changes in Doppler sounds and blood flow velocity. In this technique, the precordial Doppler monitor is placed on the left or right parasternal border at the level between the 3rd and 6th intercostal space. The monitor detects the Doppler signals derived from the patient’s intracardiac blood flow. A small amount of normal saline (NS) is then administered via a PIV line to induce changes in the Doppler signals^5,6^. The precordial Doppler can be attached during the induction of anesthesia and potentially be used throughout the surgery.

We previously used an NS injection dose of 0.5 mL/kg, based on findings obtained from a small, exploratory data set^5,6^. Therefore, the minimally required dose of NS for confirming correct PIV placement remains unknown. The repetitive intraoperative administration of NS in this precordial ultrasound technique can lead to unnecessary skin-tissue damage if PIV infiltration exists. Meanwhile, an insufficient dose of NS can increase the rate of false-negative results when attempting to confirm correct PIV placement. Thus, there is a critical need to determine the minimum amount of NS required to confirm correct PIV access placement using precordial Doppler ultrasound.

The objective of this study was to determine the minimally required dose of NS for confirming correct PIV access placement using precordial Doppler ultrasound. We hypothesized that the identification rates for changes in Doppler sound and blood flow velocity would increase with larger NS doses administered via PIV access.

## Methods

### Study design and settings

This single-center, triple-blinded, prospective interventional study was conducted at Aichi Children’s Health and Medical Center, a 200-bed tertiary care children’s hospital in Japan. The study was conducted from June 2021 to March 2022 and adhered to the Consolidated Standards of Reporting Trials (CONSORT) statement^7^. The study was approved by the institutional review board of Aichi Children’s Health and Medical Center (2020057, August 25, 2020) and was conducted in accordance with the tenets of the Declaration of Helsinki. Informed consent was obtained in all cases from the patient’s parents or guardians. The registration ID issued by the University Hospital Medical Information Network (UMIN) was UMIN000041330, date 06/08/2020 (Registry https://center6.umin.ac.jp/cgi-open-bin/icdr_e/ctr_view.cgi?recptno=R000047182).

### Inclusion and exclusion criteria

We recruited patients who underwent elective surgery and had a total body weight (TBW) between 10 and 20 kg, as well as an American Society of Anesthesiologists Physical Status (ASA-PS) score of 1 or 2. Exclusion criteria comprised the following: history of congenital heart disease, diagnosis of chromosomal abnormalities, PIV access in the lower limbs, and duplicated cases during the study period. In addition, we excluded cases in which the guardians’ primary language was not Japanese. Patients undergoing ambulatory surgery were also excluded due to the difficulty in obtaining consent before randomization in a timely manner.

### Randomization and blinding

The study participants were randomly allocated to receive one of the following three NS doses: 0.1 mL/kg, 0.3 mL/kg, and 0.5 mL/kg (simple randomization) based on the TBW. Following the sample size estimation, the research investigator (TK) created a random allocation table in a 1:1:1 ratio via STATA 17.0® (StataCorp, College Station, TX, USA). Two data managers (YS, TY) consecutively allocated the participants into the three NS dose groups using the random allocation table. The data managers (YS, TY) wrote the allocated NS dose information on a small piece of paper, which was then wrapped with aluminum foil and concealed in an envelope.

### Preparation of NS

The case-assigned anesthesiologist opened the envelope and prepared the allocated amount of NS before the study participants entered the operating suites. The amount of NS was calculated based on the patient’s TBW. The smallest possible syringe size was chosen to draw NS. The anesthesiologists were able to select either a slip-tip 2.5 mL syringe, slip-tip 5 mL syringe, or luer-lock 10 mL syringe (Terumo®, Tokyo, Japan).

### Anesthesia induction

Inhalational anesthesia was induced using 5–8% sevoflurane, and 40% N_2_O was administered for patients without preoperative PIV access. A PIV access was placed on the patient’s upper limb. Immediately after PIV access placement, several measures were used to confirm correct PIV placement and function, as well as to detect any signs of PIV infiltration or dysfunction. First, an operating nurse visually confirmed that infusion via gravity drip proceeded smoothly via the PIV access. Second, the case-assigned anesthesiologist confirmed the absence of resistance by administering a small amount of NS, followed by observation of the insertion site. Another PIV access was placed when PIV infiltration or dysfunction was detected or suspected. After the PIV access on the upper limb was secured, intravenous anesthetic medications (2 mg/kg propofol and 2 µg/kg fentanyl, with or without 0.6–1.2 mg/kg rocuronium) were injected. For cases in which a PIV had already been secured preoperatively for any reason, intravenous anesthesia was induced using the aforementioned medications; the same measures used to confirm correct PIV access and its function were performed before intravenous anesthetic injection. Once deep sedation had been achieved, either endotracheal intubation or supraglottic airway device insertion was performed, and mechanical ventilation was initiated.

### Data collection

Data collection (i.e., recording the sounds and blood flow velocity captured using the precordial Doppler machine) was performed by the investigators (AO, TK) in the operating suites after the patient’s airway was secured with an endotracheal tube or a supraglottic airway device.

All data were collected using a precordial Doppler ultrasound machine (ES-100V3®, Hadeco®, Kanagawa, Japan). Doppler sounds were recorded using a microphone (Sanwa Supply USB microphone®, Sanwa Supply®, Okayama, Japan) and recording software (Audacity 2.0®, The Audacity Team®, California, USA). The blood flow velocity was captured using a precordial Doppler machine and recorded using software (Wavetest®, Hadeco®, Kanagawa, Japan).

A precordial Doppler probe (BF8M1558A®, Hadeco®, Kanagawa, Japan) with the following properties was used: maximum intensity of < 310 W/cm^2^, intensity spatial peak temporal average of < 94 mW/cm^2^, intensity spatial peak pulse average of < 190 W/cm^2^, frequency of 2.25 MHz, and beam area of 15.7 cm^2^. The Doppler probe was fixed on the anterior chest wall using adhesive tape, with patients in the supine position. The probe location was chosen to maximize the baseline Doppler heart sound on the right or left side of the parasternal border, at the level between the 3rd and 6th intercostal space. The Doppler sounds and blood flow velocity were recorded simultaneously.

Data collection was performed using the following procedure: (1) a syringe filled with an allocated amount of NS was connected to the three-way stopcock, which was positioned at the most proximal location to the insertion site of the PIV catheter; (2) the baseline precordial Doppler sound was confirmed; (3) the baseline Doppler sound and blood flow velocity were recorded for 10 s, followed by injection of the allocated amount of NS through the three-way stopcock at the highest speed possible; and (4) the recordings were continued until 10 s after the initiation of the NS bolus. The research investigator (TK) labelled each data recording with the participant number (based on the inclusion order) provided by the two data managers (YS, TY). The data managers (YS, TY) created a correspondence table that matched the participant number and random allocation results. This correspondence table was concealed from the raters (YO, MT), research investigators (AO, TK), and data analyst (AO) until the completion of the data analysis. The data managers (YS, TY) were not involved in data collection or analysis during the study.

### Rater training

Two pediatricians (YO, MT) without knowledge of this study were recruited as raters to evaluate changes in precordial Doppler sounds. The raters were trained by research investigators (AO, TK) via previously recorded Doppler sounds with and without significant Doppler sound changes after NS injection.

### Procedures for measurement of outcomes

#### Changes in the precordial Doppler sound test (S-test)

The two raters (YO, MT) listened to the recorded Doppler sounds in a quiet location outside the operating suites; this was performed independently on different days to avoid bias. After listening to the audio files, the raters documented whether they identified a change in Doppler sounds. Research investigators (AO, TK) played the audio files, and each rater listened to the sound. The raters were blinded from the computer display that showed graphical waveforms of Doppler sound to avoid providing clues of Doppler sound changes. Changes in Doppler sounds were considered to have occurred when both raters reported a noticeable increase in pitch and volume in the S-test.

#### Changes in the blood flow velocity test (V-test)

The mean blood flow velocity was calculated for each 5-s period before and after NS injection; each 5-s period included 500 separate data measurements. Subsequently, the absolute difference between the mean velocity values before and after injection was obtained. The difference between mean velocity values was classified as positive when the difference was ≥ 1 cm/s (V-test). The cut-off value of 1 cm/s was based on the validation results of a previous study^5^.

### Outcomes

The primary outcomes were (1) the proportion of cases with a detected change in precordial Doppler sounds (S-test) and (2) the proportion of cases with a change in mean blood flow velocity of ≥ 1 cm/s in the 5-s period before and after NS injection via PIV access.

### Statistical analysis

In describing summary statistics, categorical variables are reported as numbers and percentages, while continuous variables are reported as means and standard deviations (or medians and interquartile ranges, based on the normality of the data). The proportion of cases with a positive S-test result was compared among the three dose groups using the chi-square test for rater 1 (0.1 mL/kg versus 0.3 mL/kg, 0.1 mL/kg versus 0.5 mL/kg, 0.3 mL/kg versus 0.5 mL/kg). These comparisons were only made for the results of rater 1 (YO) and not rater 2 (MT), in order to minimize the risk of Type I error inflation. For the S-test, the interrater agreement for each dose was evaluated using Cohen’s kappa statistic. The proportions of cases with positive V-test results in the three different dose groups were compared using the chi-square test. The proportions of positive test results for the S- and V-tests in each dose group were compared using McNemar’s chi-square test. For the V-test, the paired t-test was used to compare the blood flow velocity between the baseline and post-injection phase for each of the three dose groups (0.1 mg/kg, 0.3 mg/kg, and 0.5 mg/kg). Logistic regression was used to calculate the crude odds ratios (ORs) of positive S- and V-test results in the different dose groups (i.e., 0.1 mg/kg and 0.3 mg/kg using 0.5 mg/kg as the reference). Cases with missing data were excluded from the analysis. Data were analyzed using Stata 17.1 (StataCorp, College Station, TX, USA), with a two-sided *p* value of < 0.05 serving as the criterion for statistical significance. In order to correct for potential Type I error inflation, the Bonferroni correction was used for comparisons among the three NS dose groups (0.1 mL/kg versus 0.3 mL/kg, 0.1 mL/kg versus 0.5 mL/kg, 0.3 mL/kg versus 0.5 mL/kg); a two-sided *p*-value of < 0.016 served as the criterion for statistical significance.

### Sample size calculation

The proportion of positive S-tests was set at 50% for the 0.1 mL/kg dose, 70% for the 0.3 mL/kg dose, and 90% for the 0.5 mL/kg dose, based on our previous investigation^5^. The total estimated sample size was determined to be 375 (125 per group), based on the results of a chi-square test that assumed a Type I error of 0.016 and Type II error of 0.2. After allowing for a 5% data loss due to unusable or missing data, we aimed to recruit a total of 394 participants.

### Ethical declarations

Registration ID issued by the University Hospital Medical Information Network (UMIN): UMIN000041330.

Registry https://center6.umin.ac.jp/cgi-open-bin/icdr_e/ctr_view.cgi?recptno=R000047182.

Institutional review board approval number: 2020057, August 28, 2020.

The study protocol followed the tenets of the Declaration of Helsinki. There was no deviation from the original protocol throughout the study.

Written consent was obtained in all cases from the patient’s parents or guardians.

## Results

**Figure 1:**
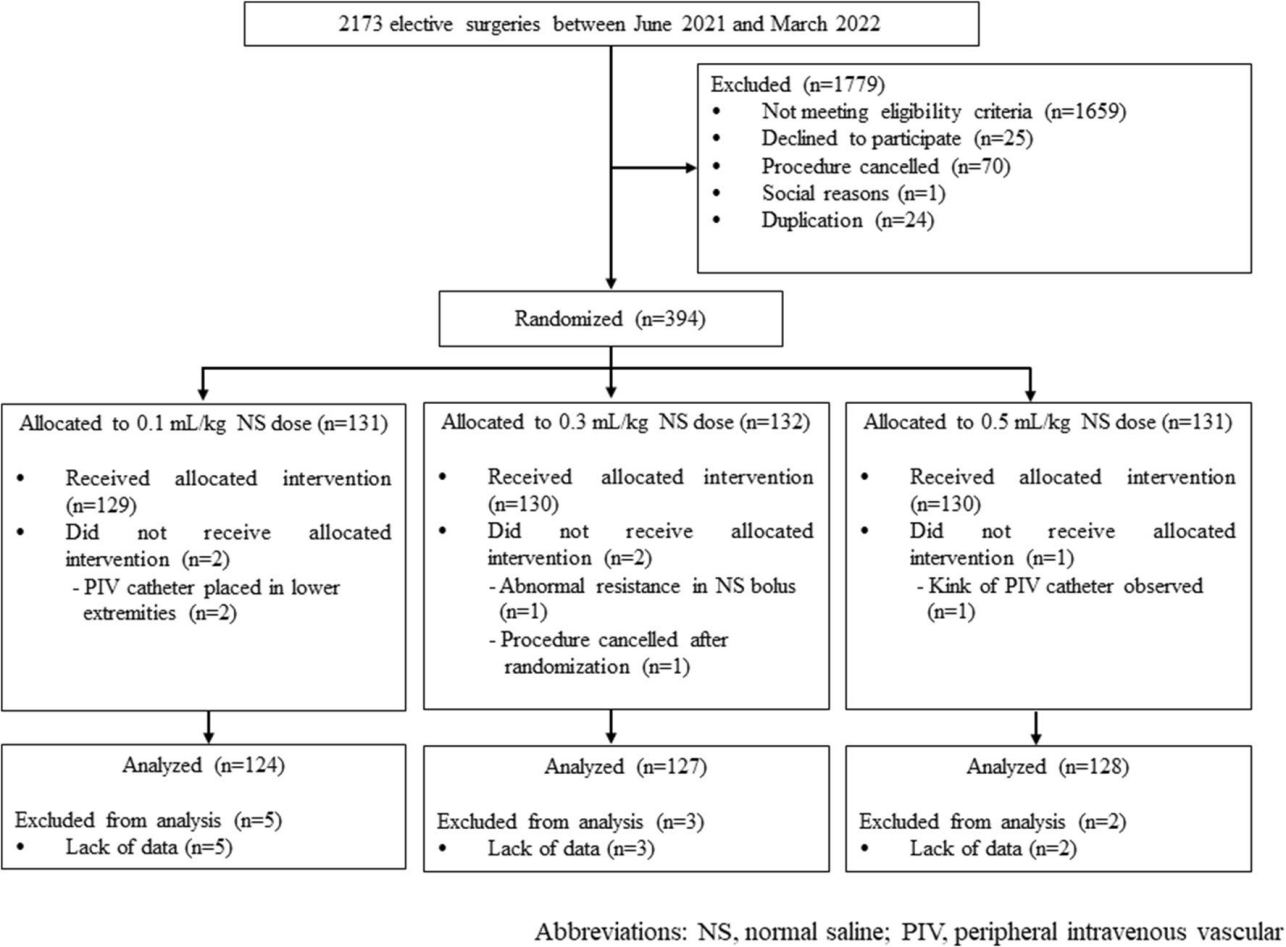
Flow diagram of participant selection. NS, normal saline; PIV, peripheral intravenous vascular.

Among 2173 patients who underwent elective surgeries, 394 patients were enrolled based on the inclusion criteria during the study period between June 1, 2021, and March 9, 2022 (Fig. 1). This trial ended after the scheduled number of participants had been enrolled.

### Patient characteristics

The demographic characteristics of each group are shown in Table 1.

**Table 1:** Patient characteristics (n = 379).

Demographic characteristics	0.1 mL/kg NS dose (n = 124)	0.3 mL/kg NS dose (n = 127)	0.5 mL/kg NS dose (n = 128)
Age, year	3.5 (2, 5)	4 (2, 5)	4 (2, 5)
Male sex (%)	71 (57.3)	78 (61.4)	70 (54.7)
Body weight (kg)	14.8 (3.1)	15.1 (3.0)	14.9 (2.8)
Body mass index (kg/m^2^)	16.0 (1.7)	15.7 (1.5)	15.9 (1.5)
ASA-PS classification (%)			
I	104 (83.8)	107 (84.3)	99 (77.3)
II	20 (16.1)	20 (15.8)	29 (22.7)
Site of PIV catheter (%)			
Cephalic vein (forearm)	14 (11.3)	8 (6.3)	14 (10.9)
Radial vein	2 (1.6)	1 (0.8)	6 (4.7)
Dorsal hand vein	108 (87.1)	118 (92.9)	108 (84.4)
Size of PIV catheter (gauge) (%)			
24	15 (12.1)	15 (11.8)	11 (8.6)
22	109 (87.9)	111 (87.4)	117 (91.4)
20	0 (0)	1 (0.8)	0 (0)
Measure of airway management (%)			
Tracheal intubation	36 (29.0)	50 (39.4)	52 (40.6)
Laryngeal mask	87 (70.2)	73 (57.5)	75 (58.6)
No device	1 (0.8)	4 (3.2)	1 (0.8)
Location of probe (%)			
Left	121 (97.6)	125 (98.4)	126 (98.4)
Right	3 (2.4)	2 (1.6)	2 (1.6)

Data presented as median (25th, 75th percentile) or No. (%).

ASA-PS, American Society of Anesthesiologists Physical Status; NS, normal saline; PIV, peripheral intravenous catheter.

Patients were between 1 and 9 years old (median, 4; interquartile range 2, 5). The PIV catheter was placed at the dorsal hand vein in 334 (88.1%) patients and the cephalic vein in the forearm in 36 (9.5%) patients. Either a 22-gauge (337 [88.9%]) or 24-gauge (41 [10.8%]) needle was used in the majority of patients.

### Inter-rater agreement (S-test)

The Cohen’s kappa value was 0.72 (*p* < 0.001) for the judgment of Doppler sound changes between the two raters across all recordings in the three dose groups. The Cohen’s kappa values for the 0.1 mL/kg, 0.3 mL/kg, and 0.5 mL/kg dose groups were 0.74 (*p* < 0.001), 0.66 (*p* < 0.001), and 0.69 (*p* < 0.001), respectively.

### Primary outcome

#### Proportion of cases with a noticeable change in Doppler sounds (S-test)

The proportions of positive S-tests for each dose group, as determined by rater 1, were 50.8% (0.1 mL/kg), 73.2% (0.3 mL/kg), and 78.1% (0.5 mL/kg) (*p* < 0.001). The proportions of positive S-tests by rater 2 were 47.6% (0.1 mL/kg), 72.4% (0.3 mL/kg), and 76.6% (0.5 mL/kg) (*p* < 0.001). A post-hoc analysis among the three dose groups, as assessed by rater 1, showed significant differences between the 0.1 mL/kg and 0.3 mL/kg dose groups (*p* < 0.001), as well as between the 0.1 mL/kg and 0.5 mL/kg dose groups (*p* < 0.001); however, there was no significant difference between the 0.3 mL/kg and 0.5 mL/kg dose groups (*p* = 0.36). The OR for detecting a Doppler signal change was significantly decreased with the 0.1 mL/kg dose versus the 0.5 mL/kg dose for both raters 1 and 2 (Table 2).

**Table 2:** Positive results in the S-test as determined by rater 1 and rater 2.

Dose (mL/kg)	Rater 1			Rater 2		
	OR	95% CI	*p* value	OR	95% CI	*p* value
0.5	1 (Reference)			1 (Reference)		
0.3	0.77	0.43–1.36	0.36	0.80	0.46–1.42	0.45
0.1	0.29	0.17–0.50	< 0.001	0.28	0.16–0.48	< 0.001

CI, confidence interval; OR, odds ratio; S-test, precordial Doppler sound test.

### Change in blood flow velocity (V-test)

The mean (standard deviation) blood flow velocity at the baseline and post-injection phase were as follows: 16.5 (4.2) and 21.3 (5.4) for 0.1 mL/kg (p < 0.001); 16.3 (3.5) and 22.6 (5.0) for 0.3 mL/kg (p < 0.001); and 16.4 (3.9) and 23.1 (4.4) for 0.5 mL/kg (p < 0.001), respectively. The proportions of positive V-tests for each dose group were 81.5% (0.1 mL/kg), 91.3% (0.3 mL/kg), and 93.0% (0.5 mL/kg) (*p* = 0.008). A post-hoc analysis among the three dose groups revealed a significant difference between the 0.1 mL/kg and 0.5 mL/kg doses (*p* = 0.008); no significant differences were observed between the 0.1 mL/kg and 0.3 mL/kg doses (*p* = 0.025) or between the 0.3 mL/kg and 0.5 mL/kg doses (*p* = 0.63). While the OR for a positive V-test result was significantly decreased for the 0.1 mL/kg dose versus the 0.5 mL/kg dose, there was no significant difference between the 0.3 mL/kg dose and the 0.5 mL/kg dose (Table 3).

**Table 3:** Positive results in the V-test for each dose of normal saline.

Dose (mL/kg)	OR	95% CI	*p* value
0.5	1 (Reference)		
0.3	0.80	0.32–2.00	0.629
0.1	0.33	0.15–0.75	0.008

CI, confidence interval; OR, odds ratio; V-test, blood flow velocity test.

### Comparison of the proportion of positive test results in the S-test and V-test for each NS dose

**Figure 2:**
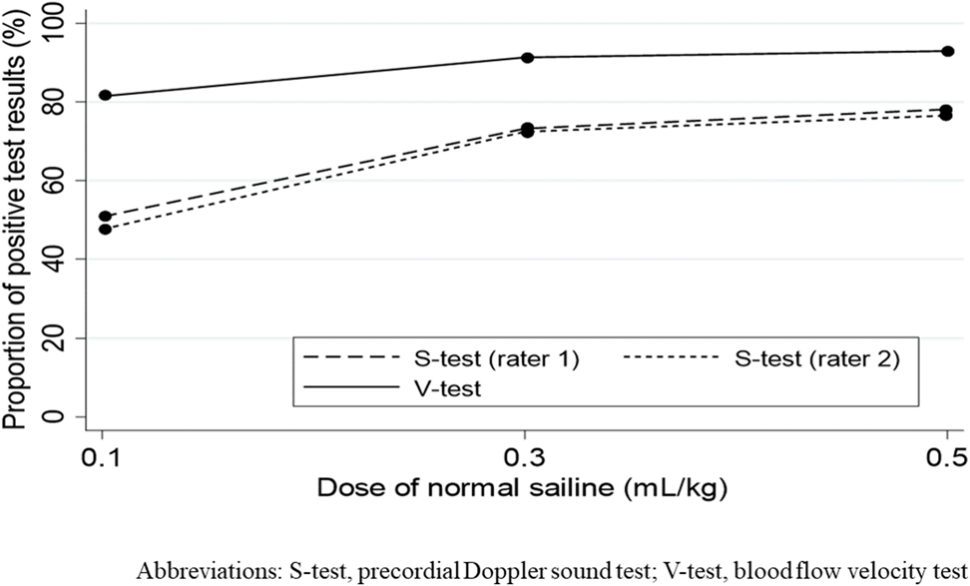
Changes in the proportion of positive test results in the S-test and V-test for different doses of normal saline. The proportions of positive test results were significantly higher in the V-test than in the S-test across all doses of normal saline. S-test, precordial Doppler sound test; V-test, blood flow velocity test.

The proportions of positive results (as per rater 1) in the S-test and V-test for each of the three doses were statistically significant in all dose groups (0.1 mL/kg, 0.3 mL/kg, 0.5 mL/kg; all *p* < 0.001) (Fig. 2).

### Harms

No adverse events were observed in this study.

## Discussion

This triple-blinded randomized trial investigated the minimally required amount of NS injection for confirming proper PIV placement via precordial Doppler ultrasound technology in children under general anesthesia. The identification rate of Doppler signal changes in both the S-test and V-test were decreased in the 0.1 mL/kg NS group. There were no significant differences in identification rates between the 0.3 mL/kg and 0.5 mL/kg dose groups, suggesting a ceiling effect for the NS dose. Despite the subjective nature of the judgments, the S-test results exhibited good inter-rater agreement, thus indicating high reliability. The V-test demonstrated a higher sensitivity than the S-test in all dose groups. The 0.3 mL/kg NS dose was considered as minimally required dose for both the S- and V-tests when confirming correct PIV placement in children.

While previous studies have reported on the utility of the precordial Doppler machine for confirming correct PIV, a detailed method has not yet been established. Precordial Doppler technology has been demonstrated to be one of the most sensitive and non-invasive tests for the detection of intravenous air emboli during craniotomy^8,9^. Our preliminary study found that the sound change on precordial Doppler ultrasound after injection of 0.5 mL/kg NS (S-test) reflected correct PIV placement with a sensitivity of 71% and specificity of 97%. Furthermore, an increase of > 1 cm/s in blood flow velocity in the V-test indicated correct PIV placement with a sensitivity and specificity of 57% and 97%, respectively. However, it remained unclear whether it was appropriate to use a 0.5 mL/kg NS dose for this purpose.

The optimal dose of NS must be investigated for several reasons. First, the amount of NS in each injection should be minimized to avoid unnecessary volume loading, as even a small amount of NS may affect a patient’s circulatory condition if multiple injections are administered, especially in patients with cardiac and renal disorders. Nevertheless, as correct PIV placement needs to be confirmed at regular intervals during surgery, multiple repeat NS injections are required during long surgical procedures. An insufficient NS dose may also increase the risk of false-negative results in both the S- and V-tests, which can result in unnecessary placement of an additional PIV access and interrupt surgical procedures.

This study compared the rates of positive results between the S- and V-tests for different NS dose groups. The V-test yielded significantly higher detection rates than the S-test in all NS dose groups. Furthermore, given its objective nature, the V-test may represent an optimal approach for confirming correct PIV access in children, provided that an NS dose of at least 0.3 mL/kg is administered. Further multi-center investigations will be required to confirm this finding. In the meantime, the V-test requires data analysis using computer software and is therefore inconvenient for intraoperative use. Further developments in hardware and software are vital for addressing this issue.

The mechanisms underlying changes in the precordial Doppler signal after NS injection are currently unclear. An earlier study presumed that the Doppler probe detects the signals created by tricuspid valve movements since Doppler sounds are often detected over the right parasternal area, just prior to and during the onset of systole^10^. A more recent study suggested that the placement of the probe on the left parasternal border detects the Doppler signal change after NS injection with a higher sensitivity than the right parasternal border^11^. As the tricuspid valve is located immediately posterior to the sternum, it is unlikely that valve movement is detected by the Doppler probe with such a high probability on the left sternal border. Therefore, we presume that the signals collected by the precordial Doppler machine reflect an influx of blood flow into the right ventricle. The baseline Doppler sounds may have been audible on the right parasternal border in some cases, most likely due to deviation of the right ventricle toward the right side as a result of anatomical changes such as atelectasis and cardiac dilatation. When microbubbles flow into the right ventricle, they may create characteristic Doppler sounds and velocity changes by enhancing Doppler signals^12^. The dose of injected NS likely affects the number of microbubbles generated, thus accounting for our finding that a higher NS dose was associated with greater detection rates for changes in precordial Doppler sounds.

Several important limitations of the present study should be acknowledged. First, the gauge of the PIV catheter may have been a confounding variable. The smaller gauge of the PIV may have slowed the injection speed, resulting in fewer microbubbles. Nevertheless, the randomization procedure should have minimized any potential residual confounding effects. Second, multiple anesthesiologists carried out NS injections during the study period. Although the research investigators instructed them to inject the NS at the highest speed possible, there may have been some variations in the injection speed. Third, the timing of the injection may not have been exactly 10 s after the initiation of the recording in either the S-test or the V-test. This small time difference may have been a source of measurement bias in the V-test. To minimize such bias, our data collection procedure specified the elapsed time for each measurement. Fourth, there may have been selection bias, as we limited our inclusion criteria to children with a body weight ranging from 10 to 20 kg and the absence of any history of congenital heart disease or any major comorbidities. Thus, further prospective studies in different populations are required. Fifth, our results lack generalizability, as the study was conducted at a single center. Sixth, the S-test applies a subjective outcome that is based on the physician’s judgment, which can cause a measuring bias. However, we used an objective outcome in the V-test (the proportion of cases with a change in the mean blood flow velocity of ≥ 1 cm/s in the 5-s period before and after NS injection via PIV access). This threshold value was determined based on a previous study^5^. The results of both the S- and V-tests showed a similar trend of a decrease in positive results (absence of change in correct PIV access) for the NS dose of 0.1 mL/kg compared with the NS dose of 0.5 mL/kg. Finally, we did not investigate the applicability of precordial Doppler ultrasound during surgery.

In conclusion, the current results indicate that the minimally required NS dose for detecting changes in precordial Doppler signals and confirming correct PIV placement in children is 0.3 mL/kg. However, further multi-center prospective investigations are needed to evaluate the applicability of this finding to other pediatric patient groups.

## Supplementary Information

Supplementary Information.

## Data Availability

The datasets generated during the current study are available from the corresponding author on reasonable request.

## Abbreviations

ASA-PS: American Society of Anesthesiologists Physical Status
CI: Confidence interval
CONSORT: Consolidated Standards of Reporting Trials
IQR: Interquartile range
NS: Normal saline
PIV: Peripheral intravenous
TBW: Total body weight
S-test: Precordial Doppler sound test
UMIN: University Hospital Medical Information Network
V-test: Blood flow velocity test
